# Shrimp hemocyanin elicits a potent humoral response in mammals and is favorable to hapten conjugation

**DOI:** 10.1038/s41598-024-67715-1

**Published:** 2024-07-22

**Authors:** Huiwen Sun, Moris Wei, Amber Guo, Ci Zhang, Yuefeng Wang, Renhui Huang, Xiaoxiao Li, Jeffrey Zhan, Jonny Wu, Bruce Jiang

**Affiliations:** 1 Shanghai Epizyme BioMedical, Shanghai, China; 2 Tongling Epizyme BioMedical, Tongling, China; 3 Shanghai Yilawo Biotech., Shanghai, China

**Keywords:** Shrimp hemocyanin, Hapten, Immunogenicity, Antibody development, Vaccine, Carrier proteins, Vaccines, Peptide vaccines

## Abstract

Conjugation to a carrier protein is essential to give rise to the antigenicity of hapten. Three carrier proteins e.g. KLH (Keyhole Limpet hemocyanin), BSA (bovine serum albumin), and OVA (Ovalbumin) were used mostly. KLH is advantageous to the others, majorly owing to its strong immunogenicity and limited usage in other biological assays. However, the cost of obtaining Keyhole Limpet is high and the solubility of KLH is not as well as the other carriers, especially after hapten conjugation. Here, we extracted the shrimp hemocyanin (SHC) from *Litopenaeus vannamei (L. vannamei),* which is a commonly sea product worldwide. The high pure SHC could be acquired by two-step purification, with a production yield of > 1 g proteins (98% pure) per 1 kg shrimp. Compared to KLH, the peptide-SHC conjugates exhibit higher solubility after hapten conjugation. Meanwhile, compared with KLH, SHC induces comparable antibody production efficiency in mammals, with or without conjugation. Furthermore, rabbit polyclonal antibodies or mouse monoclonal antibodies were generated by immunizing SHC-peptide conjugates, and the subsequent antibodies were confirmed to be used in western blot, immunofluorescence and immunohistochemistry. Therefore, we demonstrated that SHC may be used as a substitute for KLH in future antibody and vaccine development.

## Introduction

Hapten, a low molecular weight substance without immunogenicity, could stimulate immune response and antibody production when coupled to a carrier protein^1–3^. The mechanism may be explained by that hapten-carrier conjugate is able to cross-link B-cell receptors and activate T cells through the MHC class II presentation^4,5^. For the generation of antibodies against hapten or the hapten vaccine, three carrier proteins were used for covalent conjugation, e.g. KLH (Keyhole Limpet hemocyanin), BSA (bovine serum albumin) and OVA (ovalbumin)^6^. BSA and KLH could induce a strong immune response, while OVA is less effective and its solubility is not as high as the others^7^. The prevalent usage of BSA in many other biological assays has limited its use for immunization. For instance, BSA is usually used as a blocking reagent in ELISA, where anti-BSA antibody would interfere with the downstream detection. KLH is regarded as the priority for preparing hapten-carrier conjugates for immunization. However, its solubility and cost are not as well as BSA, especially after peptide conjugation. KLH is purified from the hemolymph of the inedible marine mollusc, Keyhole limpet (*Megathura crenulata)* native to the Pacific coastal waters of California and Mexico^8^. Aquaculture of Keyhole limpet is the main way to provide the animal resources for KLH extraction on a large scale, which results in the high economical cost for antibody or vaccine development. Therefore, an appropriate substitute with better biochemical characteristics, and lower economical cost, is still needed for preparing hapten-carrier conjugates.

Hemocyanins are respiratory proteins occurring freely dissolved in the hemolymph of many arthropods and molluscs^9–11^. Similar to molluscs, arthropods are evolutionally distant from mammals^12^, suggesting that hemocyanin from arthropods may induce a strong immune response in mammals. The hemocyanin from the white-leg shrimp *Litopenaeus vannamei (L. vannamei)*)^13,14^, which belongs to the phylum Arthropoda, was considered after several logical thoughts. Firstly, *L. vannamei* is a traditional marine food and its aquaculture is prevalent worldwide. According to the Food and Agriculture Organization (FAO) of the United Nations, aquaculture of *L. vannamei* contributed to 80% of the whole shrimp production, which reached 4,000,000 metric tonnes in 2014^15^. This data means the obtainment of *L. vannamei* is easy and economical. Secondly, the molecular weight of the arthropod hemocyanin subunit is 72 kDa, which is much lower than KLH (350 or 400 kDa in molecular weight)^9^. In general, lower weight means a higher solubility. Furthermore, previous studies have successfully purified the shrimp hemocyanin (SHC) from *L. vannamei* by several purification steps and demonstrated its potential application in biomedical use e.g. for tumor and microbes infection therapy^16–19^*.* However, whether hemocyanin from arthropods could be used as a carrier protein is unknown. In this study, we extracted hemocyanin from *L. vannamei* and determined its potential to serve as a peptide-conjugated carrier protein for antibody development.

## Materials and methods

### Reagents

Sulfosuccinimidyl-4-[N-maleimidomethyl] cyclohexane-1-carboxylate) (sulfo-SMCC) (*Cat.* M123456), 1-ethyl-3-(3-dimethylaminopropyl)carbodiimide hydrochloride (EDC) (*Cat.* A638729) were purchased from Aladdin (Shanghai). KLH (Cat. 77600) was purchased from ThermoFisher. Peptide synthesis was conducted by Genscript (Nanjing) in the form of dried powder. The sequence of Peptides is shown in the supplementary table.

### Preparation of shrimp serum

Healthy *Litopenaeus vannamei* with a weight between 15 and 20 g from Shanghai Didong Corporation were obtained and reared in 25-L seawater tanks at 25 °C. Air was continuously supplied using an electric pump. Hemolymph was taken directly from the pericardial sinus using a sterile tube and then allowed to clot overnight at 4 °C as described in previous study^20^. The serum was separated after centrifuging at 5000 *g* for 10 min and kept at − 20 °C until use. All animal experiments were conducted according to the recommendations from the Animal Ethics Procedures and Guidelines of the People’s Republic of China.

### Chromatographies

SHC purification was performed by gel-filtration chromatography and anion-exchange chromatography as previously described^20^. Briefly, 2 ml of *L. vannamei* serum was loaded onto a Sephadex G-100 column, and then, the column was washed with Tris–HCl buffer (0.05 M, pH 8.0) at a flow rate of 1 ml/min until the absorbance at 280 nm reached baseline. Then, the above proteins were loaded onto a DEAE-cellulose column, equilibrated with 0.01 M pH 7.5 phosphate-buffered saline (PBS) buffer. Elution was performed with 0.5 M NaCl at a flow rate of 2 ml/min. proteins concentration was determined by a nano-drop at Absorbance at 280 nm and the purity was detected by coomassie-blue (Epizyme) staining after SDS-PAGE separation.

### Peptide conjugation

Carrier protein dissolved in phosphate-buffered saline (PBS) at the concentration of 10 mg/mL was mixed with 1 mg/mL sulfo-SMCC in PBS at room temperature for 1 h. Then, the mixture was loaded on a Sephadex G25 column for desalting using 0.1 M sodium phosphate buffer, pH 7.0. Activated protein was collected according to the optical density (OD) of 280 nm and 260 nm (ratio of 280/260 > 1 because SMCC has a high absorbing density of OD260 compared to protein). Next, the peptide dissolved in PBS was mixed with the activated carrier at the final concentration of 1 mg/mL at room temperature for 2 h. For EDC conjugation to prepare peptide-conjugates in ELISA screening, 1 mg/mL EDC was mixed with 10 mg/mL BSA and 1 mg/mL peptide, which were dissolved in MES pH3.5 buffer at room temperature for 30 min.

### Protein structure prediction

SHC sequence was acquired from the website “https://www.uniprot.org/uniprotkb/G9BYP1/entry” and “https://www.uniprot.org/uniprotkb/Q26180/entry#sequences”. Alphafold-3 online server was used to simulate the tetramer of SHC. Two copies of G9BYP1 and Q26180 was set for predicting the tetramer assembly. In Fig. 2A, Chimera was used to label different colors in the SHC (G9BYP1) stimulated by alphfold-2, which could be downloaded from the alphfold database as an PDB file.

### Immunization

Rabbits were immunized by administering biweekly injections of the antigen with a total amount of 1 mg. At each immunization, the amount of antigen was same. Antigens were peptides conjugated to the indicated carriers. The injection sites on the rabbit are shaved and disinfected before immunization. Subcutaneous injection was applied at multiple injection sites, where the area between the shoulder blades (dorsal scapular region) or along the back was included. Complete Freund's adjuvant is only used with the first immunization, and incomplete Freund's adjuvant is used with the following immunization. Final boosting immunizations are performed in phosphate-buffered saline (PBS) without Incomplete Freund's adjuvant. Serum samples of 40 mL were collected after the termination of immunization, normally 30 days after the first immunization. The process for immunizing mouse is same as for rabbit, except the antigen amount. The mouse BALB/c were immunized with 10 ug antigen biweekly and the blood sample was collected at a 3-day interval. This experiment was conducted with the help of Qingdao Kangda Biotech.

### Hybridoma fusion and monoclonal antibody screening

To isolate B-lymphocyte producing certain antibodies, BALB/c female mice are immunized through repeated injection of a specific antigen as indicated above. Spleen cells were harvested from immunized mice and fused with SP2/0 myeloma cells using polyethylene glycol (PEG). The cell mixture was incubated with PEG for 1 min, followed by gradual addition of serum-free medium over 5 min. Cells were then washed and resuspended in HAT medium (hypoxanthine-aminopterin-thymidine) to select for hybridomas. The cell suspension was distributed into 96-well plates and incubated at 37 °C with 5% CO2. After 10–14 days, hybridoma growth was assessed, and supernatants were screened for antibody production using ELISA. Positive clones were expanded and subcloned by limiting dilution to ensure monoclonality.

All experimental procedures and methods employed in this study were conducted in strict accordance with the relevant guidelines and regulations of Epizyme Biomedical. Ethical approval was obtained from the appropriate institutional review board, ensuring that all protocols adhered to the ethical standards and regulatory requirements.

### Cell culture

HEK293T, HepG2, HeLa and other cells as indicated were cultured in DMEM (Dulbecco's Modified Eagle Medium) supplemented with 10% fetal bovine serum (FBS) and 1% penicillin–streptomycin. Cells were maintained at 37 °C in a humidified atmosphere with 5% CO2. Cells were passaged at 70–80% confluence by washing with PBS, detaching with 0.25% trypsin–EDTA, and reseeding at a 1:5 to 1:10 dilution. Medium was replaced every 2–3 days.

### Western blot analysis

Western blot analysis was performed as previously described^9^. Briefly, HEK293T, HepG2, HeLa and other cells were collected in a loading buffer (Epizyme) after one wash with PBS. Of note, harvest was conducted at almost 85% cell confluence. Total proteins were separated by SDS polyacrylamide gel electrophoresis and transferred onto an NC membrane. Coomassie blue (Epizyme) staining was used to visualize the total cellular protein and ensure they were equivalent (data not shown). The membrane was blocked using 5% dried milk and incubated with the indicated primary antibody and secondary antibody. Bands were visualized using Omni ECL reagent (EpiZyme) under an illuminating imager XF101 (Epizyme), and the grey intensity was acquired by using Fiji (NCBI). Serum harvested from the non-immunized animals was used as a negative control, and no band could be detected (data not shown).

### Immunohistochemistry (IHC)

Tissue was fixed with formaldehyde for 3 h at room temperature, then dehydrated at a sequential concentration of ethanol and embedded in paraffin. The samples were then sectioned at a thickness of 5 to 20 μm and paraffin was removed by xylene and hydrated at a sequential concentration of ethanol. Antigen retrieval was by heat mediation in Tris pH 9. Samples were blocked with 1% BSA and incubated with primary antibody for 16 h at 4 ℃. An HRP-conjugated goat anti-rabbit IgG polyclonal was used as the secondary antibody. DAB was added to develop the signal for 1–5 min until the brown color in the sample. The remaining DAB was removed by water several times, the nuclei were further stained by Hematoxylin. The sample was mounted in a resin for photography. The serum from non-immunized rabbit or mouse was used for a negative control for IHC and no specific signal could be detected.

### Immunofluorescence

Cells were fixed with 4% paraformaldehyde for 5 min and penetrated with 0.05% TrionX-100 for 2 min at room temperature. Then, 10% FBS in PBS were used for blocking, and the primary antibody was treated for 1 h, followed by the FITC-conjugated goat anti-mouse IgG at a dilution of 1:5000 for 1 h. Nuclei were counterstained with DAPI for 1 min. The image was captured by KEYENCE BZ-X800LE through DAPI and GFP channels and data were generated by deconvolution process. Of note, a negative control was set without the addition of the primary antibody.

### Electron-microscopy (EM)

EM was performed as describe previously^21^. In brief, 3ul protein (10 ug/uL) dissolved in 1 × PBS was dropped on a piece of para-film, and a carbon-coated nickel grid was placed on the drop for 30–60 min. The sample was treated with 2% uranyl acetate for 30 s, washed three times with water. The grid was air-dried and disinfected under UV (ultra-violet) before examination by FEI F20 TEM at 120 kV.

### ELISA assay

Serum samples were collected, stored at − 20 °C, thawed, and centrifuged at 10,000 × *g* for 10 min. Wells of a 96-well plate were coated with 100 µL of antigen (1 µg/mL in carbonate-bicarbonate buffer, pH 9.6) and incubated overnight at 4 °C. The wells were washed with PBST (PBS + 0.05% Tween-20) and blocked with 200 µL of 5% BSA in PBS for 1 h at room temperature. After washing, 100 µL of rabbit serum (diluted 1:1000 in PBST + 1% BSA) was added to each well and incubated for 2 h at room temperature. Wells were washed and incubated with 100 µL of HRP-conjugated anti-rabbit or anti-mouse IgG (diluted 1:5000 in PBST + 1% BSA) for 1 h at room temperature. Following further washes, 100 µL of TMB substrate was added and incubated for 15–30 min, then 50 µL of 0.1 M HCl was added to stop the reaction. Absorbance was measured at 450 nm using a microplate reader.

### Statistical analysis

The continuous variables in different subgroups were compared using an unpaired *t*-test and a one-way analysis of variance. All the tests were two-sided, and p < 0.05 was considered statistically significant. The statistical analyses were performed using GraphPad.

## Results

### High pure SHC was acquired from *L*. *vannamei* by two-step chromatography

**Figure 1:**
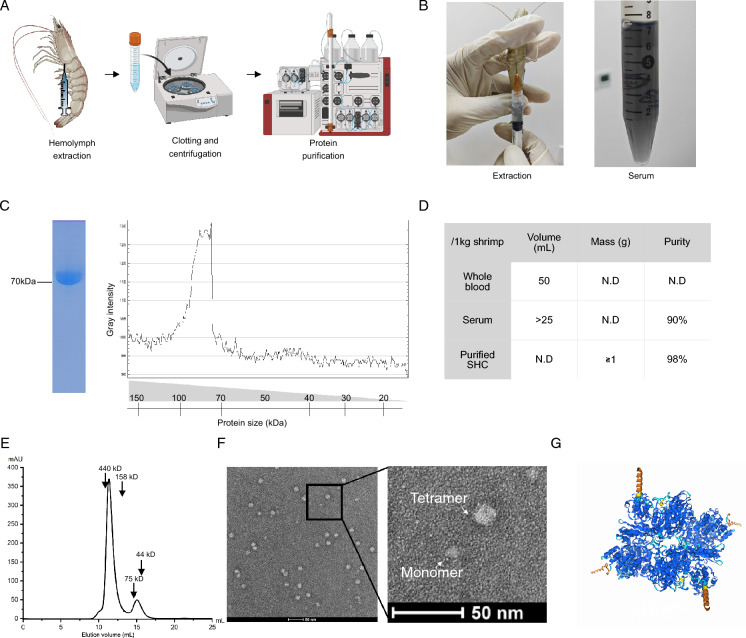
SHC extraction and purification from *L. vannamei.* (**A**) Schematic representation of the SHC purification. (**B**) Photos of hemolymph and serum extracted from shrimps. A blue-black color could be observed. (**C**) SDS-PAGE separation of purified SHC. Proteins were detected by Coomassie-blue staining. Fiji software was used to acquire the grey intensity in the gel. (**D)** A calculation of the SHC yield and purity at the indicated steps. *N.D*. represents not determined. (**E**) Size exclusion chromatography of purified SHC. SHC was dissolved in PBS and loaded into Superdex-200 column. Size was determined by running of standard proteins representing different molecular size. (**F**) Electron-microscopy analysis of purified SHC. (**G**) Alphafold-3 prediction of SHC protein. Two copies of 74.9 or 76.6 kDa SHC subunits were given to the Alphafold-3 program for simulation. A screenshot was acquired from the online Alphafold-3 server.

Adult *L. vannamei* obtained from the market was applied to hemolymph extraction. Then, serum containing SHC was collected after the centrifugation of clotted hemolymph and SHC was further purified by chromatography (Fig. 1A). One shrimp is about 10 g in weight on average, which could provide 0.5 mL of whole hemolymph after extraction through the pericardial sinus region (Fig. 1B). The hemolymph and serum showed a clear black-blue color, indicating that SHC is rich in the liquid (Fig. 1B). In addition, SHC could be visualized by coomassie-blue staining after SDS-PAGE separation, where a band with a molecular weight close to 70 kDa was observed (supplementary Fig. 1A). Next, we conducted the purification via size exclusion and ion exchange chromatography. After the two-step purification, SHC with 98% purity is acquired (Fig. 1C,D). On the other hand, the solubility of SHC is about 200 mg/mL at the neutral pH condition, which is comparable to its counterpart KLH (supplementary Fig. 1B). Furthermore, size-exclusion chromatography analysis showed that 80% of SHC were between 158 to 440 kDa in weight, while the remaining between 44 to 75 kDa (Fig. 1E), suggesting that SHC preferred to form polymers in physical environment. Consistent with this, we observed that most proteins were nearly 20 nm in diameter and a small amount of them were less than 10 nm under transmission electron microscope (Fig. 1F), which corresponded to the diameter of the monomer or tetramer simulated by Alpha-fold2 based on the primary amino-acids sequence (Fig. 1G). Overall, we could postulated that SHC is able to polymerize to form the tetramer in-vitro. Altogether, the production efficiency of high pure SHC protein is more than 1 g per 1 kg shrimps, highlighting the possibility of large-scale SHC production for commercial usage.

### SHC is superior to KLH in solubility when conjugated to peptides

**Figure 2:**
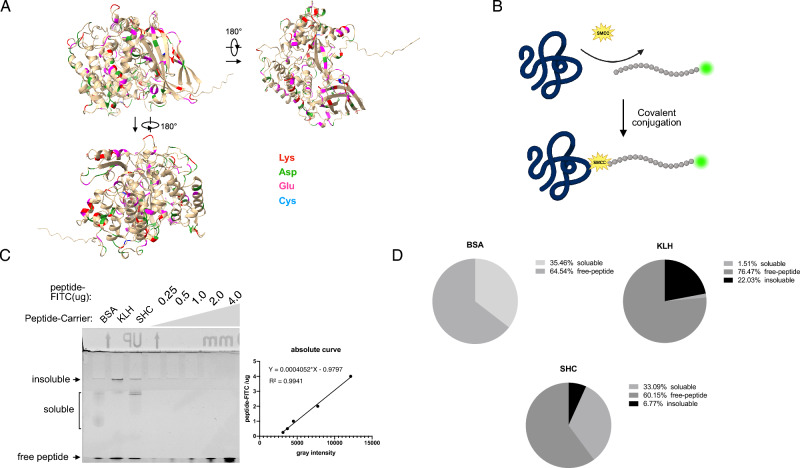
Generation of SHC-peptide conjugates. (**A**) 3D structure of SHC proteins predicted by Alphafold-2. (**B**) Schematic representation of SMCC-mediated covalent linkage of peptide to carrier proteins. (**C**) SDS-PAGE gel separation of peptide-carrier conjugates. A serial dilution of FITC-peptide was used to make the standard curve for the absolute determination of conjugates. Of note, a large complex that stayed in the sample well was defined as the insoluble conjugates, which were turbid in reaction liquid. (**D**) Statistics of conjugation efficiency and conjugates solubility.

Among 672 amino acids (Supplementary Fig. 2A) in SHC, there are 36 Lys (K), 57 Asp (D), 47 Glu (E) and 2 Cys (C). Thus, besides the terminal -NH3 and -COOH, one SHC contains 36 -NH3, 104 -COOH and 2 -SH. Alpha-fold2 structural simulation of SHC indicated that there are adequate Lys, Asp and Glu exposed on the surface (Fig. 2A), which may be accessible for hapten linkage. We applied a conjugation assay to test the conjugation efficiency of SHC, where FITC-labelled peptide and SMCC linker were explored (Fig. 2B). In the neutral pH environment, SMCC activates -NH3 in the donor, and the SMCC-activated SHC was desalted by a Sephadex G25 column (Supplementary Fig. 2B). The activated amine then selectively attacks -SH in the recipient and forms a covalent bond (Fig. 2B). Of note, in the reaction mix, 1 mg/mL SMCC-activated carriers (BSA, 15.3 μM; SHC, 13.8 μM; KLH, 2.8 μM) were mixed with 1 mg/mL FITC-peptide (1.68 kDa, 618 μM) in their final concentration. After the conjugation, 2.5 uL conjugates were loaded into SDS-PAGE gel. Signals were detected by the fluorescence via a 488 nm filter (Fig. 2C). BSA, KLH and SHC formed the covalent bond to FITC-labelled peptide with different binding efficiency. About 35.46% of peptides conjugated to the BSA were soluble (Fig. 2C,D). About 33.09% of peptides linked to SHC were soluble and 6.77% were insoluble. In contrast, insoluble KLH-peptide conjugates dominate, as only 1.51% were soluble. Including soluble and insoluble conjugates, the conjugation efficiency of BSA, SHC and KLH was calculated as 35.46%, 39.86%, and 23.54%, respectively, suggesting that one molecule of BSA, SHC and KLH is able to link approximately 14, 17 and 59 peptide molecules at average, respectively. In principle, one SHC may link 36 peptide molecules. This difference between theoretical prediction and experimental results may result from the steric hindrance or the protein cross-linking. Nevertheless, these data suggested that SHC is suitable for hapten conjugation and its solubility is superior to KLH after peptide conjugation.

### The immunogenicity of SHC is potent in mammals compared with KLH

**Figure 3:**
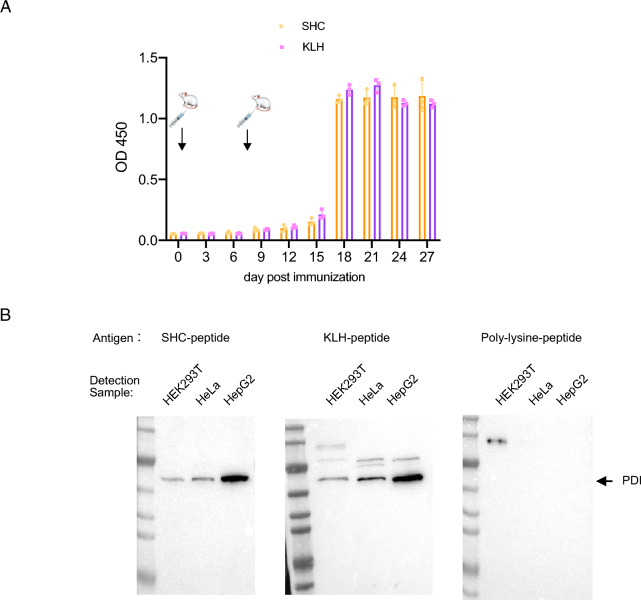
SHC owns the comparable immunogenicity to KLH. (**A**) Titer determination of humoral response after mice immunized by SHC and KLH. In each group, 3 female mice were used for immunization at the indicated time in figure. ELISA was used to detect the level of anti-SHC or anti-KLH antibodies. (**B**) SHC-peptide stimulated the production of rabbit antibodies against the PDI gene. Equal cell numbers were loaded for WB detection.

To determine the immunogenicity of SHC in mammals, equal amounts of KLH and SHC were given to BALB/c mice. Blood sample were harvested every three days and the titer of anti-KLH or anti-SHC were determined by ELISA assay. Humoral antibody response could be detected fourteen days post the first immunization, while strong antibody production against KLH or SHC was seen thirty days post the first immunization (Fig. 3A). No significant difference in antibody titers could be observed between KLH and SHC immunized groups. Furthermore, rabbits were immunized by three carriers (KLH, SHC and poly-lysine) conjugated to the peptide designed for the PDI gene. Poly-lysine was set as a negative control as it generally does not induce a strong immune response in mammals. The rabbit serum was collected and applied to WB analysis, where cell-line samples were used as the native antigen (Fig. 3B). Note that equal cells were loaded and no difference in the mass of whole cellular proteins was observable as confirmed by coomassie-blue staining (data not shown). Consistency with the transcriptional level of PDI mRNA (supplementary Fig. 3), a similar protein expression pattern of PDI was detected in HEK293T, HeLa and HepG2 by the serum from rabbits immunized with KLH or SHC conjugates. Together, we demonstrated that SHC owns the potent immunogenicity compared with KLH.

### Confirmation of SHC extensiveness for rabbit polyclonal antibody development

**Figure 4:**
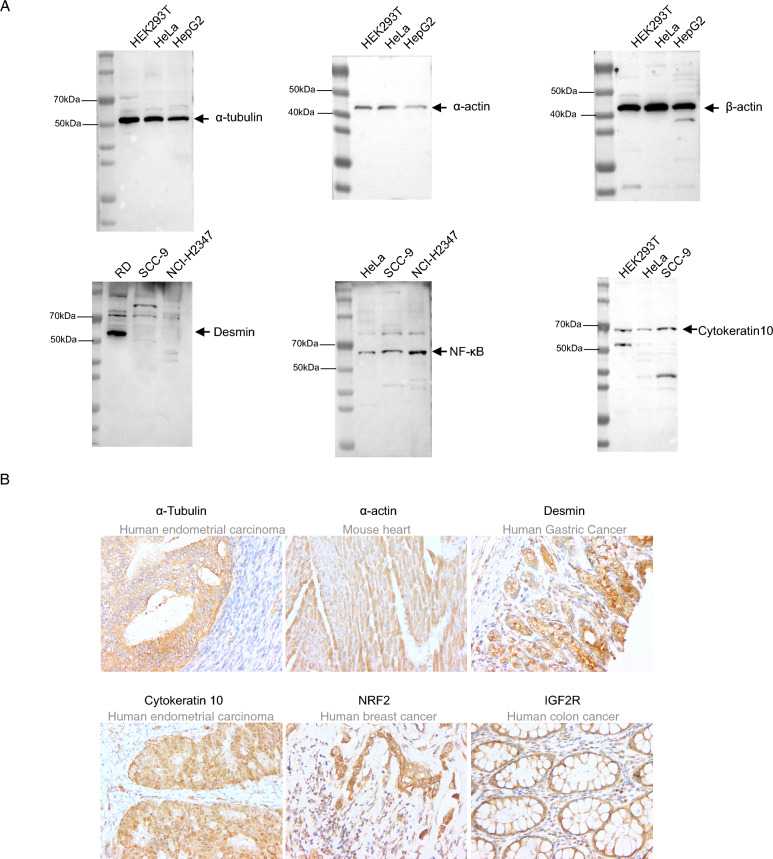
SHC could be used for rabbit polyclonal antibody development. Peptides targeting the indicated proteins were designed and conjugated to SHC. Rabbit serum was harvested 30 days after the first immunization. (**A**) Confirmation of the rabbit serum application in WB detection. Of note, equal cell numbers were loaded. (**B**) Confirmation of the rabbit serum application in IHC detection.

To extend the use of SHC for hapten immunization, SHC-peptide conjugates were prepared for generating polyclonal antibody against the following several genes: α-tubulin, α-actin, β-actin, Desmin, NF-κB, Cytokeratin 10, NRF2, IGF2R, most of which are cellular host genes or the common targets in biological research. WB and IHC were conducted to test the rabbit serum harvested thirty days after the first immunization. The rabbit sera against α-tubulin, β-actin, Desmin, and Cytokeratin-10 were applicable in both WB and IHC detection, while sera against others were applicable for at least one of the two assays (Fig. 4A,B). WB data showed that the bands of cellular host genes were clear and antibody specificity was high. Although detection results of Desmin, NF-kB and Cytokertin-10 showed some un-specific bands, the specific bands are present in the correct molecular weight and the expression pattern was in consistence with their RNA transcription level (Supplementary Fig. 4). On the other side, the positive signal in IHC analysis showed the correct morphological distribution of the corresponding antigen in tested organs (Fig. 4B). Of note, no signal in control without the addition of primary serum could be detected (data not shown).

### Confirmation of SHC for application in mouse monoclonal antibody development

**Figure 5:**
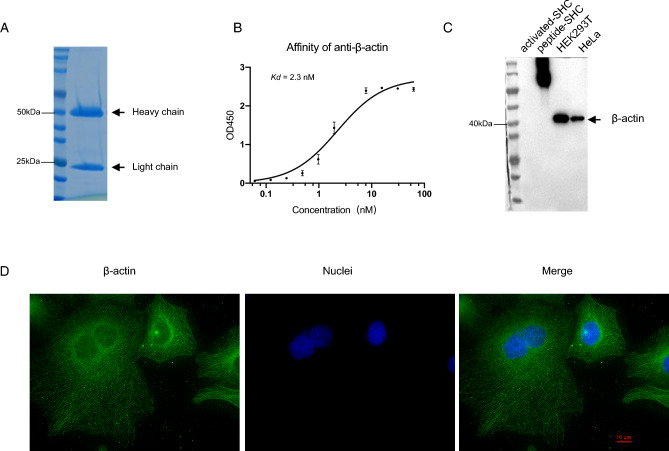
SHC could be used for mouse monoclonal antibody development. Actin peptide was cross-linked to SHC for immunization. (**A**) Protein G column purification of anti-β-actin from hybridoma supernatant. The purified antibody was detected by SDS-PAGE separation. (**B**) Determination of dissociation constant (Kd) of peptide and anti-β-actin. Peptide-BSA conjugates were coated in 96-well, then a serial dilution of purified anti-β-actin were incubated as the primary antibody. All the other steps were performed as same as the ELISA assay. The Non-linear fit by one-site specific binding in GraphPad Prism 8 was applied to calculate the Kd value. R^2^ = 0.9345. (**C**) Confirmation of the application of anti-β-actin in WB detection. Peptide-SHC conjugates were set as a control. Equal cell numbers were loaded. (**D**) Confirmation of the anti-β-actin application in IF detection. β-actin was visualized by the green while nuclei were visualized by blue. HeLa was used for this detection.

Next, we asked whether SHC could be applied in monoclonal antibody development. After immunization of peptide-SHC conjugates for anti-β-actin generation in BALB/c mouse, spleen were sectioned and splenocyte was isolated by ficol density centrifugation. PEG1450 were used for the fusion of Sp2/0 and splenocyte. Several positive clones were finally screened by Elias assays. One clone anti-β-actin (4B6) was purified from the hybridoma supernatant by protein G purification with the affinity of Kd = 2.3 nM (Fig. 5A,B). This 4B6 clone was further confirmed in WB application, where a cellular actin band close to 40 kDa was visualized (Fig. 5C). Filament-like morphology of actin in cytoplasm was clearly observed by immunofluorescence (Fig. 5D). Together, these data demonstrated the applicability of SHC in monoclonal antibody development.

## Discussion

Carrier proteins play a vital role in the generation of hapten-related antibodies and vaccines because hapten is not able to be processed by antigen-presenting cells with itself alone. Nowadays, KLH is the most commonly utilized carrier due to its strong immunogenicity and limited application in biochemical assays. Obtainment of KLH mainly relies on the aquaculture of the sea animal keyhole limpet, which is generally inedible worldwide. Moreover, the solubility of KLH is low after conjugation, resulting the difficulty in the downstream purification. Here, we determined that SHC possesses comparable immunogenicity to KLH while owning a higher solubility after conjugation. SHC is extracted from the most popular seafood, the white-leg shrimp or *L. vannamei* in the Latin name. In our hand, from 1 kg healthy shrimp, 50 mL hemolymph could be extracted and 1 g high-pure SHC could be acquired finally. This high yield could pave the way for SHC industrialization. In future, SHC would reduce the research and production costs related to vaccine and antibody development.

Several reasons may explain why the solubility of SHC-peptide conjugates is higher than that of KLH conjugates. Firstly, the molecular weight of SHC is much lower. SHC is 74.9 kDa or 76.6 kDa in the molecular weight, whereas KLH is 350 or 400 kDa. Secondly, using bioinformatic analysis of the amino-acids sequence, we found that SHC is more thermally stable than KLH (Supplementary Table 3). Thirdly, compared to KLH, the ultrastructure of SHC complexes is simpler. Arthropod hemocyanins form hexamers and their multiples, whereas mollusk hemocyanins form decamers or larger aggregates with a more complex arrangement of functional units^22^.

Except for *L. vannamei,* other crustaceans e.g. Argentine red shrimp (*Pleoticus muelleri*), Red swamp crawfish (*Procambarus clarkii*), Chinese mitten crab (*Eriocheir sinensis*), Giant tiger prawn (*Penaeus monodon*) etc.^15^, also provide the resource for hemocyanin extraction. These crustaceans could be captured from wild areas or harvested by aquaculture. In this study, we selected the *L. vannamei,* given that this species dominates in the crustacean production worldwide. The high production of *L. vannamei* would guarantee the sustainable supply of shrimp hemocyanin at a relatively low expense. Furthermore, after hemolymph extraction, *L. vannamei* could be processed as a by-product such as dried shrimp. Therefore, SHC is more economical than KLH.

In antibody development, SHC would be more suitable for hapten-conjugation than KLH considering the solubility. However, in vaccine research^23^, especially for preparing human vaccines, one thing should be considered. *L. vannamei* is the most common seafood and is often put on the table, whether the anti-SHC antibody is present in the human body is not known. Pre-existing antibodies against SHC in people may reduce the vaccine potency or even induce allergy^24^. On the other side, the molecular diversity of SHC may result in the nuance of production batch, which may affect the immune response against SHC and the efficacy of SHC-based vaccine^19^. Therefore, more studies are needed for applying SHC in vaccine research.

## Patent information

The utilization of SHC in hapten-carrier preparation has been submitted to patent application. The patent application numbers in Republic of China were CN2023115993853, CN2024100341885. PCT patent number was PCT/CN2024/071375.

### Supplementary Information

Supplementary Figures.

Supplementary Table 1.

Supplementary Table 2.

Supplementary Table 3.

Supplementary Legends.

## Data Availability

All data generated or analysed during this study are included in this published article and its supplementary information files.
