# Supplementary figures and images for: Shrimp hemocyanin elicits a potent humoral response in mammals and is favorable to hapten conjugation
Source: Sci Rep. 2024 Jul 22;14:16771. doi: 10.1038/s41598-024-67715-1 (PMC11263335; doi:10.1038/s41598-024-67715-1)

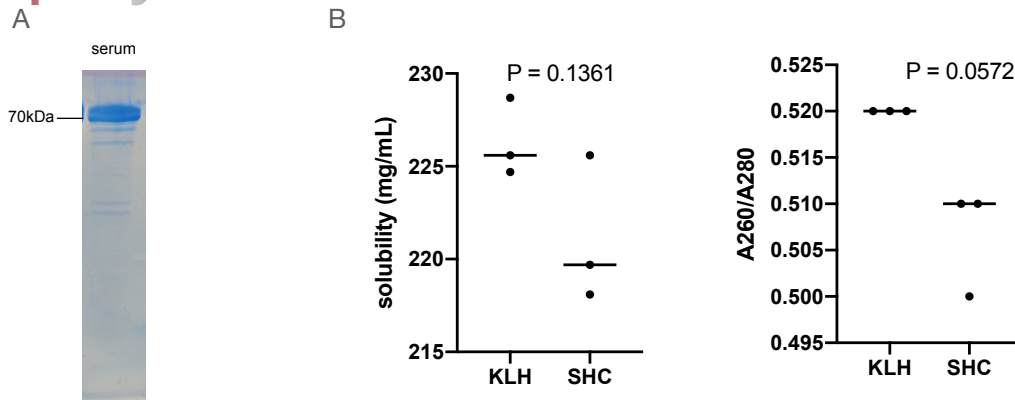

Supplementary Figure1

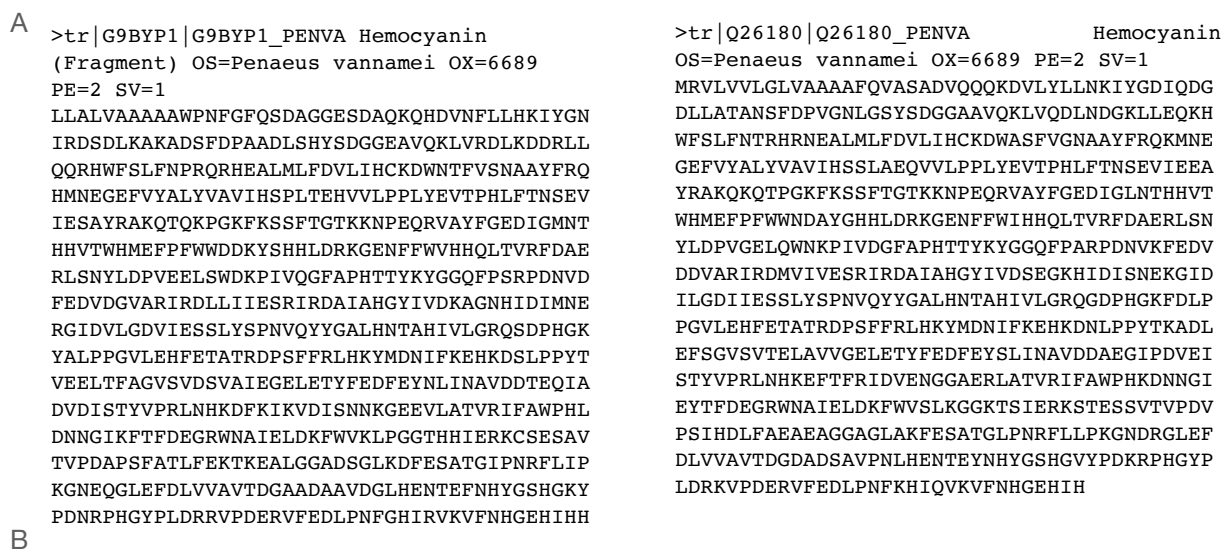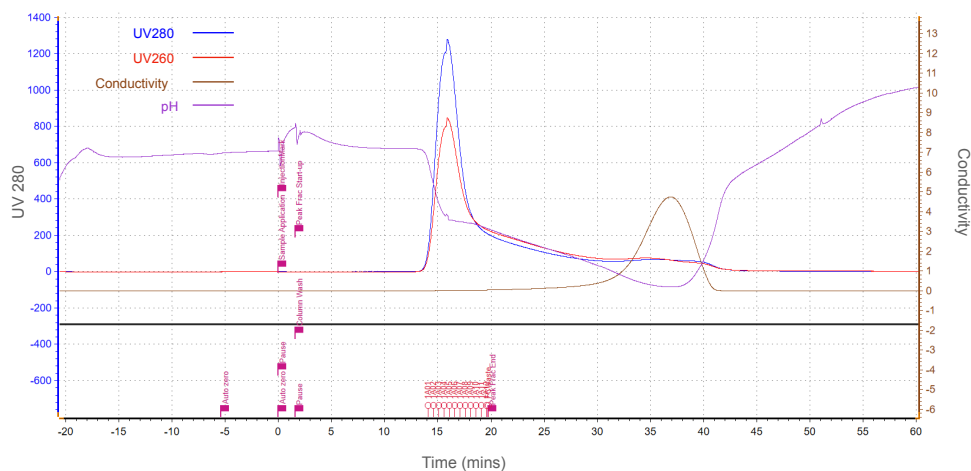

Supplementary Figure2

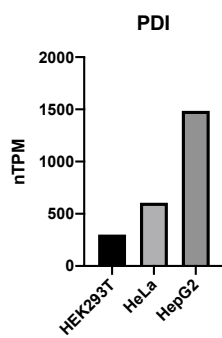

Supplementary Figure 3

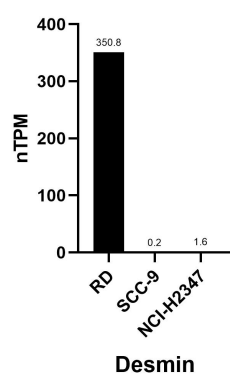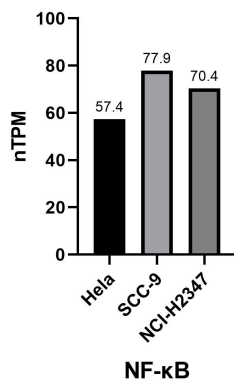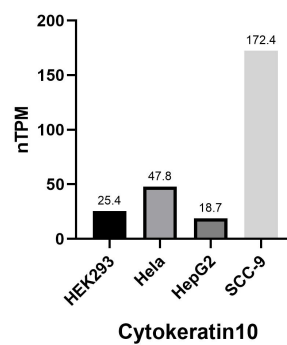

Supplementary Figure 4

Supplement: Supplementary file 1 — Supplementary Figures. [file 41598_2024_67715_MOESM1_ESM.pdf]
