# Supplementary material for: Shrimp hemocyanin elicits a potent humoral response in mammals and is favorable to hapten conjugation
Source: Sci Rep. 2024 Jul 22;14:16771. doi: 10.1038/s41598-024-67715-1 (PMC11263335; doi:10.1038/s41598-024-67715-1)
Supplement: Supplementary file 2 — Supplementary Table 1. [file 41598_2024_67715_MOESM2_ESM.pdf]

Protein/Gene name	Uniport	sequence	Position	g-score value			
PDI	P07237	EEAEEPDMEEDDDQC	488-502	7.21			
Cytokeratin 10	P13645	RYSSSKHYSSSRSGC	4-18	7.93			
Desmin	P17661	NFRETSPEQRGSEVC	427-441	6.56			
Alpha Tubulin	P68363	DGQMPSDKTIGGGDC	33-47	7.38			
NF-κB p65	Q04206	QRPPDPAPAPLGAPC	508-522	6.49			
Beta Actin	P60709	QKDSYVGDEAQSKRC	49-63	7.12			
smooth muscle act	P62736	RPRHQGVMVGMGQKC	39-53	5.91			
IGF2R-Specific	P11717	LPPPRQGKEGQENGC	2376-23[illegible]	11.69			
NRF2, NFE2L2	Q16236	LRDEDGKPYSPSEYC	568-582	7.62			
