# Supplementary material for: Shrimp hemocyanin elicits a potent humoral response in mammals and is favorable to hapten conjugation
Source: Sci Rep. 2024 Jul 22;14:16771. doi: 10.1038/s41598-024-67715-1 (PMC11263335; doi:10.1038/s41598-024-67715-1)
Supplement: Supplementary file 3 — Supplementary Table 2. [file 41598_2024_67715_MOESM3_ESM.pdf]

| Reagent | company/source | Cat.No. |
|---|---|---|
| Keyhole Limpet Hemocyanin | Thermo Fisher Scientific | 77600 |
| Bovine Serum Albumin | Aladdin | B265993 |
| Ovalbumin | Thermo Fisher Scientific | 77120 |
| Complete Freund's adjuvant | Sigma-Aldrich | F5881 |
| Incomplete Freund's adjuvant | Sigma-Aldrich | F5506 |
| Sulfosuccinimidyl-4-[N-maleimidomethyl] cyclohexane-1-carboxylate | Aladdin | M123456 |
| 1-ethyl-3-(3-dimethylaminopropyl) carbodiimide hydrochloride | Aladdin | E106172 |
| phosphate-buffered saline | Epizyme Biotech | CB012 |
| Polyethylene glycol | Sigma-Aldrich | P7181 |
| Loading buffer | Epizyme Biotech | LT103 |
| Omni ECL reagent | Epizyme Biotech | SQ202 |
| Formaldehyde | Sinopharm Chemical Reagent | 10010018 |
| Ethyl alcohol | Macklin | E809061 |
| Xylene | Sinopharm Chemical Reagent | 10023418 |
| HRP-conjugated goat anti-rabbit IgG | Epizyme Biotech | LF102 |
| DAB | Boster Biological Technology | SA2025 |
| Hematoxylin | Baso | BA4040 |
| Triton™X-100 | Aladdin | T109026 |
| Fetal Bovine Serum Premium | Epizyme Biotech | CY103 |
| FITC-conjugated goat anti-mouse IgG | Boster Biological Technology | BA1101 |
| DAPI | Sigma-Aldrich | D9542 |
| FITC-Peptide | Genscript | |
| Poly-lysine | Solarbio | P8120 |
| α-tubulin polyantibody | homo-made | |
| α-actin polyantibody | homo-made | |
| β-actin polyantibody | homo-made | |
| Desmin polyantibody | homo-made | |
| NF-κB polyantibody | homo-made | |
| Cytokeratin10 polyantibody | homo-made | |
| NRF2 polyantibody | homo-made | |
| IGF2R polyantibody | homo-made | |
| β-actin monoantibody | homo-made | |
| HRP-conjugated goat anti-mouse IgG | Epizyme Biotech | LF101 |
