# Supplementary material for: Shrimp hemocyanin elicits a potent humoral response in mammals and is favorable to hapten conjugation
Source: Sci Rep. 2024 Jul 22;14:16771. doi: 10.1038/s41598-024-67715-1 (PMC11263335; doi:10.1038/s41598-024-67715-1)
Supplement: Supplementary file 4 — Supplementary Table 3. [file 41598_2024_67715_MOESM4_ESM.pdf]

Protein	Uniprot entry name	Instability index	Aliphatic index	Grand average of hydropathicity (GRAVY)	pI
KLH1	Q10583	42.01 (unstable)	74.80	-0.460	5.87
KLH2	Q10584	42.90 (unstable)	73.21	-0.460	5.73
SHC1	G9BYP1	33.49 (stable)	77.77	-0.494	5.44
SHC2	Q26180	30.11 (stable)	82.01	-0.412	5.31

The Grand Average of Hydropathicity (GRAVY) index is a measure of the overall hydrophobicity or hydrophilicity of a protein.The GRAVY index can give insight into the protein's solubility, with positive values indicating a hydrophobic (less soluble) protein and negative values indicating a hydrophilic (more soluble) protein. These prediction was conducted by using Protparam.
