# Supplementary material for: Shrimp hemocyanin elicits a potent humoral response in mammals and is favorable to hapten conjugation
Source: Sci Rep. 2024 Jul 22;14:16771. doi: 10.1038/s41598-024-67715-1 (PMC11263335; doi:10.1038/s41598-024-67715-1)
Supplement: Supplementary file 5 — Supplementary Legends. [file 41598_2024_67715_MOESM5_ESM.docx]

Supplementary figure1. (A) Coomassie-blue staining of the serum from freshly-harvested hemolymph. (B) Left: Solubility test of KLH and SHC. A insufficient volume of 1× PBS was used to re-suspend the dried powder of KLH and SHC, where un-resolved protein was removed by centrifugation at 12,000g, 5min and supernatant was loaded to nano-drop for determining its concentration by using absorbance at 280 nm. Right: The ratio of A260/A280 was shown.

Supplementary figure2. (A) Amino-acid sequence of SHC. The sequence was acquired from Uniprot. (B) Sephadex G-25 desalting of activated SHC.

Supplementary figure3. The mRNA level of PDI in the indicated cell-line. The data was acquired from the Protein Atlas.

Supplementary figure4. The mRNA level of Design, NF-kB and cytokeratin10 in the indicated cell-line. The data was acquired from the Protein Atlas.
